# Featured Papers in *NeuroSci*

**DOI:** 10.3390/neurosci4020010

**Published:** 2023-04-30

**Authors:** Xavier Gallart-Palau

**Affiliations:** 1 Biomedical Research Institute of Lleida Dr. Pifarré Foundation (IRB Lleida), Neuroscience Area, +Pec Proteomics Research Group (+PPRG), University Hospital Arnau de Vilanova (HUAV), 25198 Lleida, Spain; xgallart@irblleida.cat; Tel.: +34-973702201; 2 Faculty of Medicine, University of Lleida, 25198 Lleida, Spain; 3 Department of Psychology, University of Lleida, 25198 Lleida, Spain

In this topical collection, Arsiwalla et al. discuss the viability of the morphospace construct to unite artificial intelligence concepts with biological agents to analyze and explore consciousness [1]. In the same vein, Pereira et al. discuss what makes neuronal organoids sentient from the perspective of sentiomics and the potential involvement of brain stimulation [2]. Liwicki et al. assess the replicability of subject-dependent and -independent methods of inner speech decoding [3].

Kieran Greer proposes an evolutionary model to explain the neural correlates that may sustain intelligence and adaptation, from invertebrates to the human brain [4]. Using comparative neuroscience, Peguero et al. investigate how neuronal tissue from zebrafish can be maintained ex vivo and how it responds molecularly to neuronal insult, with applicability in human neuronal regeneration [5]. Ison et al. use crustacean neurons to investigate the effects of Doxapram on neuronal channels, a platform that can potentially be extrapolated to investigate the effects of several other drugs on neuronal channels [6]. Carvalho et al. explore the alcohol addictive effects in rats that have experienced early life stressful events [7]. Kiffmeier et al. analyze the sex-dependent implications of the cerebellum on the learning process in autism model BTBR mice [8].

Relevant studies involving human subjects and specific clinical populations have also been included in this book. Valdez et al. investigate single neuron response to human face image processing [9], dePace et al. investigate autonomic neuropathies [10], Lentoor et al. explore neurocognitive domains in obesity [11], Gallo et al. look at bilateral facial palsy at the onset of neurosarcoidosis [12], Colombo et al. analyze the neuromodulation of dysautonomia in long COVID patients [13], Tan el al. assess the safety of the use of fluorescein sodium in pediatric neurosurgery [14], and Tariciotti et al. design a deep learning model with clinical applicability in brain tumor classification [15]. Finally, Handle et al. perform an interesting literature review to identify the most relevant findings linking handwriting product patterns to the specific cognitive and behavioral idiosyncrasies of subjects affected by autism spectrum disorder [16].
